# Performance of large language models in neonatal resuscitation assessments versus healthcare providers: an exploratory study

**DOI:** 10.3389/frai.2026.1838877

**Published:** 2026-06-25

**Authors:** Chenguang Xu, Yihua Chen, Shelley Skelding, Dianna Wang, Qianshen Zhang, Georg M. Schmölzer, Po-Yin Cheung

**Affiliations:** 1 NICU, The University of Hong Kong-Shenzhen Hospital, Shenzhen, China; 2 Shenzhen University Medical School, Shenzhen, China; 3 NICU, University of Alberta, Edmonton, AB, Canada; 4 Centre for the Studies of Asphyxia and Resuscitation, Neonatal Research Unit, Royal Alexandra Hospital, Edmonton, AB, Canada

**Keywords:** large language model, neonatal resuscitation, healthcare professional (HCP), examination questions, language concordance

## Abstract

**Background:**

Artificial intelligence and large language models (LLMs) have developed rapidly in recent years and involved in medical education, in addition to clinical care. However, it remains unknown how the performance of LLMs in neonatal resuscitation compares to that of healthcare professionals (HCPs). In this exploratory study, we aimed to investigate and compare the performance of LLMs with those of HCPs on 3 sources of examination questions in the neonatal resuscitation training in Canada and China.

**Methods:**

In this bi-center study, we evaluated the overall accuracy, accuracy across question types, and reliability of LLMs’ (ChatGPT-5 and DeepSeek-R1) responses to neonatal resuscitation questions from workshop in China, NRP® textbook (8^th^ edition), and Kahoot quizzes in Canada. Each LLM was tested three times per question with one-week intervals in August and September, 2025. Comparisons with historical scores of HCPs in the training courses in China (2022–2024) and Canada (2022–2025) were performed.

**Results:**

Both LLMs performed comparably to HCPs in Chinese examinations, and showed similar accuracy in NRP® textbook questions with higher scores on multiple-choice than on short answer questions. ChatGPT achieved higher accuracy than DeepSeek and HCPs in the Kahoot quizzes. ChatGPT also had higher accuracy on scenario-based than on non-scenario-based questions in the workshop examination. High reliability of LLMs’ responses was found (Fleiss’ Kappa>0.89).

**Conclusion:**

Both ChatGPT and DeepSeek achieved accuracy comparable to that of HCPs and showed high consistency on selected neonatal resuscitation written examinations. The findings warrant further research to explore their potential integration in neonatal resuscitation training.

## Introduction

The limited training opportunities and rapid decay of neonatal resuscitation knowledge and skills among healthcare providers (HCPs) after training represent critical barriers in neonatal resuscitation training programs (Sihota et al., 2025). Large language models (LLMs) have the potential to learn from vast medical corpora and thus may offer a solution for providing educational opportunities (Goh et al., 2024; Demirtas et al., 2025; Singhal et al., 2023).

Despite the growing integration into various medical domains, it remains unknown how the performance of LLMs in neonatal resuscitation assessment compares to that of HCPs. This knowledge gap hinders the ability to determine their safe and effective role in education and clinical practice. In addition, LLMs might exhibit linguistic strength based on their primary language of training, achieving concordance in other languages might be a challenge (Dzuali et al., 2024). In languages with limited training data, LLMs are prone to filling knowledge gaps by generating fabricated information, a phenomenon known as artificial intelligence (AI) hallucination. The persuasive but inaccurate, or biased output carries substantial risk of harm (Singhal et al., 2023).

In this exploratory study, we aimed to investigate and compare the capability of LLMs with those of participants (all HCPs) in the neonatal resuscitation training in Canada and China. We assessed the performance of ChatGPT and DeepSeek in three sources of neonatal resuscitation examinations in English and Chinese languages. We tested the hypothesis that the accuracy of both LLMs would be comparable to that of HCPs on the selected neonatal resuscitation written examinations.

## Methods

### Study design, testing LLMs and population

**Figure 1:**
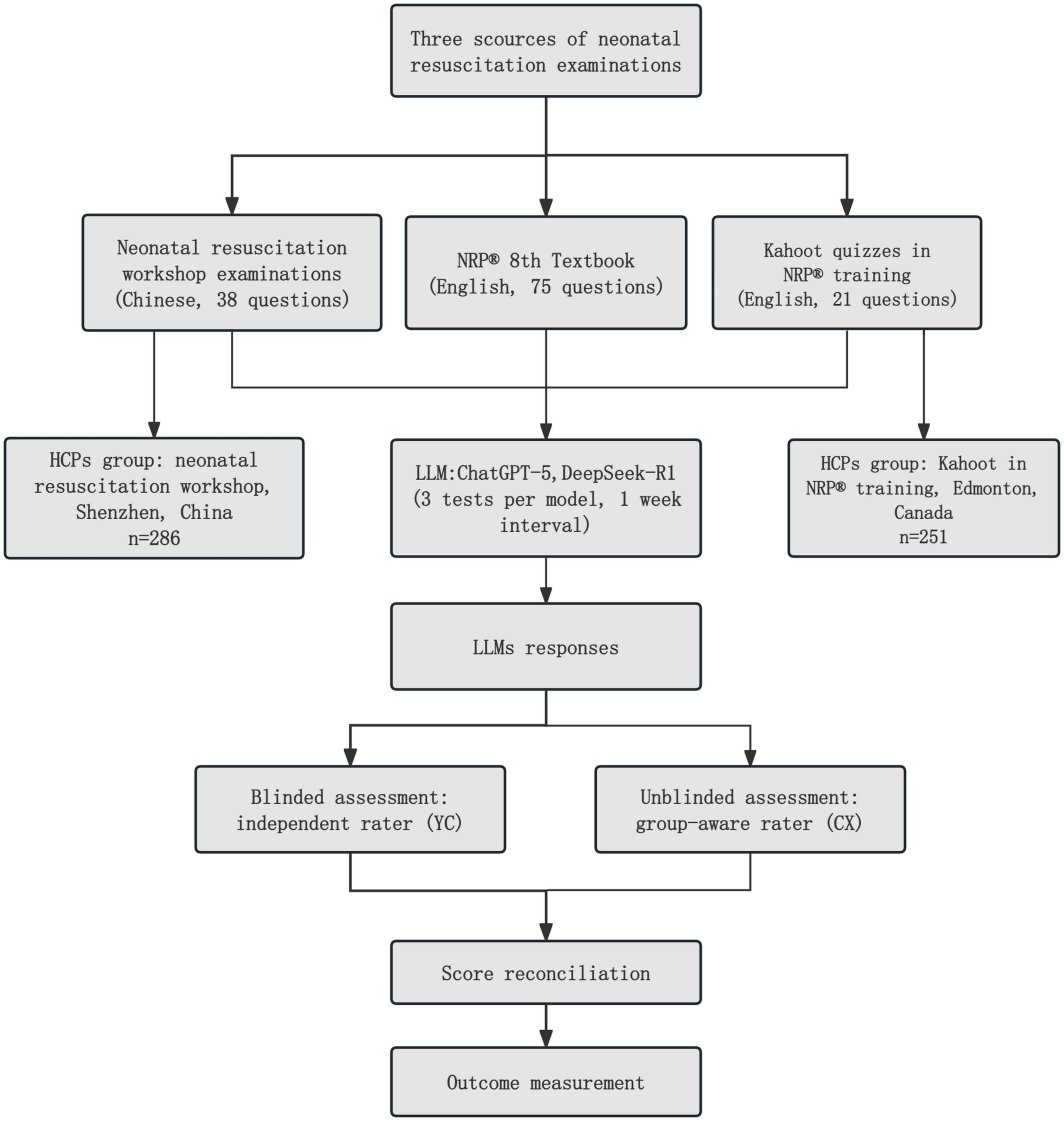
Study design and evaluation process.

This bi-center comparative study evaluated the performance of ChatGPT and DeepSeek on neonatal resuscitation questions from neonatal resuscitation workshop in China (38 questions, Chinese), the Neonatal Resuscitation Program® (NRP®) 8th edition textbook (75 questions, English) (American Academy of Pediatrics, 2021), and Kahoot quizzes in Canada (21 questions, English). Both the workshop examinations and Kahoot quizzes were designed by the institutional educators (CX and QZ, SK and DW, respectively, and other clinicians in the local neonatal resuscitation training team) in accordance with the NRP® 8th edition (American Academy of Pediatrics, 2021) (Figure 1). All questions including images were excluded. Scenario-based questions were defined as those set within a specific clinical scenario or context. These questions assessed clinical reasoning, decision-making and application of knowledge, as opposed to non-scenario-based questions which tested isolated facts or direct recalls of knowledge (Supplementary material).

ChatGPT-5 (via the website, released in August 2025, OpenAI, San Francisco, CA, United States) and DeepSeek-R1 (via API, 671B parameters, R1-0528 version, June 2025 update, DeepSeek, Hangzhou, China) were used. Browsing and retrieval were disabled. Each LLM was tested three times with one-week intervals in August and September 2025. Questions were posed in their original language using simple prompt: “Please answer the following questions.” Chain-of-thought or multi-step reasoning was provided. In each of three separate testing sessions, every query was submitted once without repetition. A new conversation session was started without any prior chat history.

The scores of 286 HCPs in the neonatal resuscitation workshop conducted by the University of Hong Kong-Shenzhen Hospital, China (2022–2024), and Kahoot scores of 251 HCPs in NRP® training in Royal Alexandra Hospital, University of Alberta, Canada (2022–2025) were reviewed. The Kahoot HCP cohort completed the quizzes as part of the NRP® training, after the didactic lecture and before the simulation training.

### Assessment of LLM responses

LLM response was scored in binary as correct or incorrect. For short answer questions, any response that was incorrect or incomplete was considered incorrect. Two neonatologists (YC, CX) independently evaluated all LLM responses using pre-defined scoring criteria. YC was blinded to response source, while CX was involved in LLM response collection and was aware of the source. Discrepancies were resolved through final cross-check.

### Outcome measures

The primary outcome was overall accuracy (expressed as percentage of number of correct answers/total number of answers). The secondary outcomes included reliability of LLMs’ responses, accuracy for scenario-based, multiple-choice and short answer questions.

### Statements of ethics

This study was reviewed and approved by the Institutional Review Board of the University of Hong Kong-Shenzhen Hospital (#hkuszh2025063) and the Institutional Review Board of the University of Alberta (#Pro00156941). Written informed consent was obtained from participants in the neonatal resuscitation workshops held at the University of Hong Kong-Shenzhen Hospital to participate in the study. For the anonymous Kahoot scores, the Institutional Review Board of the University of Alberta granted an exemption from requiring written informed consent for the use of data.

### Statistical analysis

Differences in accuracy between groups (ChatGPT, DeepSeek, and HCPs) were assessed using Chi-square test or Fisher’s exact test. In addition to analyzing the accuracy of LLMs in all questions in repeated sessions (results in Supplementary material), to avoid clustering bias from three correlated runs per question, we aggregated the three binary outcomes (correct/incorrect) using majority voting. A question was scored as correct if at least two of three runs were correct. This yielded one binary outcome per model per question. The inter-rater agreement on three responses generated by LLMs was evaluated using Fleiss’ Kappa. SPSS Statistics (v.27) was used for data analyses. A *p* value less than 0.05 was considered statistically significant.

## Results

Agreement between two assessors was high, with differences observed in 6 out of 402 answers. Discrepancies were resolved after cross-check, and a final binary outcome was agreed for each response.

### Performance comparison between LLMs and HCPs

In the neonatal resuscitation workshop examinations, ChatGPT and DeepSeek showed similar accuracy rates (90 and 92%, *p* = 0.692) (Table 1). HCPs significantly improved the scores after training (92% vs. 82% of pre-training, *p* < 0.005) but both pre-training and post-training scores were comparable to those of LLMs. In the Kahoot quizzes, ChatGPT had the highest accuracy rate (95%), followed by DeepSeek (81%) and HCPs (78%) but the differences were not significant (Table 1 and Supplementary material).

**Table 1:** Comparison of performance between large language models and healthcare providers (HCPs).

Assessments	Overall accuracy % (n/N)^#^	*p*-value		
		vs. DeepSeek-R1	vs. HCPs *pre-training* 81.9% (8,343/10184) *N* = 268	vs. HCPs *post-training* 91.7% (4,702/5130) *N* = 135
Neonatal resuscitation workshop examinations (Chinese)	ChatGPT-5 89.5% (34/38)	0.692	0.227	0.628
	DeepSeek-R1 92.1% (35/38)	—	0.103	0.921
Kahoot quizzes (English)		vs. DeepSeek-R1	vs. HCPs 78.3% (4,128/5271) *N* = 251	
	ChatGPT-5 95.2% (20/21)	0.343	0.064	
	DeepSeek-R1 81.0% (17/21)	—	1.000	

^#^: n/N represents the percentage of total correct answers over the total number of questions.

### Performance across question types

ChatGPT achieved higher scores in scenario-based than in non-scenario-based questions in neonatal resuscitation workshop (100% vs. 83%), but the difference was not statistically significant (Table 2 and Supplementary material). DeepSeek and HCPs had similar accuracy rates. ChatGPT and DeepSeek showed similar accuracy in NRP® 8th edition textbook questions. Both LLMs scored higher on multiple-choice than on short answer questions (Table 2).

**Table 2:** Comparison of performance between large language models and healthcare providers by question format, model, and data source.

Model or Data source	Overall accuracy, % (n/N)^a^	Scenario-based questions, % (n/N)^a^	Non scenario-based Questions, % (n/N)^a^	*p*-value
ChatGPT-5				
Neonatal resuscitation workshop examination	89.5% (34/38)	100% (14/14)	83.3% (20/24)	0.276
NRP® 8th textbook	93.3% (70/75)	90.5% (19/21)	94.4% (51/54)	0.645
Kahoot quizzes^a,b^	95.2% (20/21)	100% (1/1)	95.0% (19/20)	b
DeepSeek-R1				
Neonatal resuscitation workshop examination	92.1% (35/38)	92.9% (13/14)	91.7% (22/24)	1.000
NRP® 8^th^ textbook	90.7% (68/75)	90.5% (19/21)	90.7% (49/54)	1.000
Kahoot quizzes^a,b^	81.0% (17/21)	100% (1/1)	80% (16/20)	b
Healthcare providers (HCPs)				
Neonatal resuscitation workshop examination (135 HCPs)	91.7% (4,702/5130)	92.1% (1739/1890)	91.5% (2,963/3240)	0.484
Kahoot quizzes^b^ (251 HCPs)	78.3% (4,128/5271)	85.3% (214/251)	78.0% (3,914/5020)	b

NRP® 8^th^ Textbook	Overall accuracy, % (n/N)^a^	Multiple-choice questions, % (n/N)^a^	Short answer questions, % (n/N)^a^	*p*-value
ChatGPT-5	93.3% (70/75)	97.0% (64/66)	66.7% (6/9)*	0.011
DeepSeek-R1	90.7% (68/75)	95.5% (63/66)	55.6% (5/9)*	0.003

^a^: n/N represents the percentage of total correct answers over the total number of questions.

^b^: No comparison was performed because there was only one scenario-based question in the Kahoot quiz.

### Accuracy and reliability analysis

The reliability of LLMs’ responses for all examinations was high (Fleiss’ Kappa values all ≥0.90, *p* < 0.001) (Table 3).

**Table 3:** Accuracy and reliability analysis of large language models across repeated tests.

Data source	Model	Test 1 (%)	Test 2 (%)	Test 3 (%)	Fleiss’ Kappa (95% CI)	*p*-value
NRP® 8^th^ Textbook	ChatGPT	95.3	96.0	96.7	0.985 (0.978–0.991)	<0.001
	DeepSeek	93.3	95.3	92.7	0.941 (0.914–0.962)	<0.001
Neonatal resuscitation workshop examinations	ChatGPT	86.8	89.5	92.1	0.988 (0.980–0.993)	<0.001
	DeepSeek	94.7	92.1	89.5	0.898 (0.833–0.942)	<0.001
Kahoot quizzes	ChatGPT	90.5	95.2	95.2	0.900 (0.807–0.955)	<0.001
	DeepSeek	71.4	85.7	76.2	0.953 (0.905–0.979)	<0.001

### Language concordance and AI hallucination

Simple calculation mistakes and cross-lingual inconsistency were noted in some answers by both LLMs. The performance of two LLMs varied across epinephrine and flush volume and was influenced by language. When prompted in English, ChatGPT tended to provide the correct answer, whereas DeepSeek answered more correctly in Chinese. For the question on epinephrine flush volume in English Kahoot quizzes, ChatGPT consistently answered correctly, whereas DeepSeek consistently answered incorrectly in three runs. In contrast, for the same question presented in Chinese workshop examinations, DeepSeek answered correctly in all three runs, while ChatGPT provided incorrect answers in two of the three runs (Supplementary material).

## Discussion

This study suggests potential strength of LLMs in selected neonatal resuscitation written examinations with performance comparable to HCPs by rigorous, bilingual comparisons. Our results indicate that LLMs perform well in overall accuracy and scenario-based problem-solving in neonatal resuscitation, with higher scores on multiple-choice than on short answer questions.

LLMs use deep learning to understand and generate output. They excel at tasks involving language comprehension, contextual reasoning, and synthesizing information based on artificial neural networks trained on vast datasets (Tangsrivimol et al., 2025). Scenario-based questions are fundamentally reasoning tasks. They are designed to evaluate the ability to synthesize theoretical knowledge and apply it to a specific clinical context. This requires integrating multiple variables, recognizing clues, and eliminating interfering factors to arrive at a valid conclusion. This form of contextualized reasoning aligns precisely with the strength of LLMs. However, LLMs might have been exposed to neonatal resuscitation concepts, as this knowledge is found in publicly available materials including NRP® textbook and journals. Their high accuracy rates might also reflect training data contamination, leading to memorization rather than true reasoning.

LLMs’ responses are stochastic. In evaluations of LLMs on life support exams, overall accuracy varied based on the model version and testing methodology. An earlier version of ChatGPT (version:9) failed to pass the American Heart Association (AHA) Basic Life Support (BLS) and Advanced Cardiovascular Life Support (ACLS) exams in a single-attempt setting, scoring 64–68% on BLS and 68.4–76.3% on ACLS exam (Fijačko et al., 2023). When three responses per question were generated and evaluated, ChatGPT achieved an overall accuracy of 84% on BLS exam and 78.95% on ACLS exam (Zhu et al., 2023). ChatGPT-4o achieved a 90.7% overall accuracy and exceeding all the passing score on all attempts on the Pediatric Advanced Life Support (PALS) exam (Kokulu et al., 2024), while in a recent study by Demirtas et al., it attained 75.3% on the NRP® exam over three attempts (Demirtas et al., 2025). To capture a broader spectrum of potential variation and more accurately assess real-world stability, we adopted repeated measuring over long intervals. This approach is designed to account for a wider range of fluctuation sources and thus provides a more reliable measure of real-world performance. We found that ChatGPT-5 achieved higher accuracy than models in previous studies, which might be attributable to additional training and iterative advancement of the model.

Beyond resuscitation, a growing body of research has also investigated LLMs in pediatrics and medical education (Mansoor et al., 2025; Huang et al., 2026; Suresh and Misra, 2024). Mansoor et al. reported that DeepSeek-R1 achieved an accuracy exceeding the typical first-time pass rates of the American Board of Pediatrics (Mansoor et al., 2025). However, LLM’s performance on multiple-choice questions should not be equated with superior clinical competence, as they have not been tested on history taking, physical examination, or real-world management planning (Mansoor et al., 2025). We collected data from HCPs retrospectively from previous training under time constraints and real-time feedback, whereas LLMs were tested in a untimed environment. Therefore, this was not a direct head-to-head comparison but an exploratory analysis comparing the performance of LLMs against historical data from HCPs. Direct comparisons are confounded by differences in testing environment, time pressure, and presence of feedback, possible distractions, and motivational context. High accuracy of LLMs on static written questions does not equate to clinical competence (Huang et al., 2026). Nevertheless, LLMs can pass standardized examinations and may serve as effective tools for question generation, personalized study plans, and immediate feedback (Suresh and Misra, 2024). Further research should explore how LLMs can optimally enhance HCP’s learning efficiency.

LLMs exhibit relatively poor performance on simple arithmetic tasks such as single-digit addition (Goodell et al., 2025). A study reported that ChatGPT was an unreliable clinical calculator, with only a 66% accuracy (Goodell et al., 2025). Errors in simple medication dosage calculations were also identified in our study. This inadequacy poses a substantial obstacle to the implementation of LLMs, as their outputs require careful oversight in critical tasks such as determining the dosage and concentration of neonatal resuscitation medications.

Across one Chinese and two English tests, both ChatGPT and DeepSeek showed good overall accuracy. Interestingly, for the same question with identical content in Chinese and English, we observed that each LLM performed better in its dominant training language. This pattern aligns with the concept of language concordance, where LLMs tend to perform better when prompted in their dominant training language. Reasoning errors may arise when the prompt language differs from the model’s internal processing language (Strasser et al., 2026). In practice, educators need to consider model choice and prompt language to optimize its output.

Whether the advantages of LLMs could be translated into clinical practice and medical education also depend on the way they are used. While ChatGPT-4 had superior independent diagnostic reasoning capability compared to physicians, its use by physicians in facilitating diagnosis had not significantly improved diagnostic performance (Goh et al., 2024). However, ChatGPT-generated structured feedback significantly enhanced medical students’ clinical decision-making skills (Brügge et al., 2024). This indicates that simply using LLMs as information tools might fail to leverage their reasoning potential. A recent pediatric simulation study reported that LLM could generate real-time debrief scripts from simulation transcripts, improving discussion organization and learner engagement (Hong et al., 2025). Such applications suggest that LLMs may be useful in neonatal resuscitation training, where structured feedback and scenario-based learning are essential. However, further research is needed to assess LLMs’ performance in real-world settings before recommending their integration into neonatal resuscitation education.

## Limitations

Our study has several limitations. First, training examinations including questions in the neonatal resuscitation workshops and NRP® provider courses could be stressful to some HCPs because the scores are important in the certification process. Second, Kahoot is a gamified, interactive platform but it has a countdown for each question. The pressure of time might have affected the ability of some HCPs to retrieve known information or forced them to make quick choices. Therefore, the performance of HCPs might be underestimated in these situations. Further, the professional background of HCPs was heterogeneous, including NICU doctors, NICU nurses, pediatricians, respiratory therapists, midwives, and obstetricians, with a wide range of clinical experience. We were unable to further stratify and compare the performance of subgroups with LLMs.

## Conclusion

In this exploratory study, both ChatGPT and DeepSeek achieved accuracy comparable to that of HCPs and showed high consistency on selected neonatal resuscitation written examinations. The findings warrant further research to explore their potential integration in neonatal resuscitation training.

## Data Availability

The raw data supporting the conclusions of this article will be made available by the authors, without undue reservation.
