# Correction: Performance of large language models in neonatal resuscitation assessments versus healthcare providers: an exploratory study

**DOI:** 10.3389/frai.2026.1934012

**Published:** 2026-07-22

**Authors:** Frontiers Production Office

**Affiliations:** Frontiers Media SA, Lausanne, Switzerland

**Keywords:** large language model, neonatal resuscitation, healthcare professional (HCP), examination questions, language concordance

**Supplementary Table S1** was omitted. The file(s) have now been published.

The original version of this article has been updated.

