# Supplementary material for: Performance of large language models in neonatal resuscitation assessments versus healthcare providers: an exploratory study
Source: Front Artif Intell. 2026 Jun 25;9:1838877. doi: 10.3389/frai.2026.1838877 (PMC13346239; doi:10.3389/frai.2026.1838877)
Supplement: Supplementary file 1 [file Data_Sheet_1.DOCX]

**Sample questions and LLM sample answers**

1. **NRP**^®^ **8^th^ Textbook questions**

***1.1 Short answer question***

**Sample question:**

What are the 4 pre-birth questions to ask the obstetric provider before every birth?

**Suggested answer**:

The 4 pre-birth questions are: (1) What is the expected gestational age? (2)Is the amniotic fluid clear? (3) Are there any additional risk factors? (4) What is our umbilical cord management plan?

**ChatGPT answer (August 20):**

4 pre-birth questions:

· What is the expected gestational age?

· Is the amniotic fluid clear?

· How many babies are expected?

· Are there any additional risk factors?

**Comment:** The ChatGPT answer did not include umbilical cord management plan. The response was considered incomplete and was marked as incorrect.

**DeepSeek answer (August 20):**

What are the 4 pre-birth questions to ask the obstetric provider before every birth?

**•** What is the Expected Gestational Age?

**•** Is the Amniotic Fluid Clear? (or meconium-stained?)

**•** How Many Babies Are Expected?

**•** Are There Any Additional Risk Factors?

**Comment:** The DeepSeek answer also did not include umbilical cord management plan. The response was considered incomplete and was marked as incorrect.

***1.2 Scenario-based question***

**Sample question:**

Your team is resuscitating a baby born at term. The baby's heart rate is 40 beats per minute after 30 seconds of ventilation through an endotracheal tube and an additional 60 seconds of coordinated chest compressions and ventilation using 100% oxygen. You determine that epinephrine is indicated. Your team should

1. quickly attempt to insert a peripheral intravenous catheter in the baby's right hand.
2. insert an umbilical venous catheter.

**Suggested answer**: B

***1.3 Non-scenario-based question***

**Sample question:**

Intravenous epinephrine should be administered

1. slowly, followed by a 3-mL normal saline flush.
2. as quickly as possible, followed by a 3-mL normal saline flush.
3. as quickly as possible, followed by a 1-mL normal saline flush.
4. slowly, followed by a 1-mL normal saline flush.

**Suggested answer**: B

1. **Kahoot quizzes**
   1. ***Ture or False question***

**Sample question:**

A pulse oximeter is recommended prior to starting supplemental oxygen for central cyanosis. 🞎 True 🞎 False

**Suggested answer**: 🗹 True

- 1. ***Scenario-based question***

**Sample question:**

You are resuscitating a non-vigorous infant and the heart rate is difﬁcult to auscultate. What should you do?

🞎 Intubate the baby

🞎 Connect a saturation probe and/or ECG monitor

🞎 Start compressions

🞎 Check peripheral pulses

**Suggested answer**: 🗹 Connect a saturation probe and/or ECG monitor

- 1. ***Non-scenario-based question***

**Sample question:**

What is the recommended ﬂush volume for Epinephrine IV/IO?

🞎 1ml

🞎 Enough to clear the line

🞎 0.5ml

🞎 3ml

**Suggested answer**: 🗹 3ml

**ChatGPT answer (September 1, 8, 15):**

What is the recommended ﬂush volume for Epinephrine IV/IO?
🗹 *3 ml*

**DeepSeek answer (September 1, 8, 15):**

🗹 enough to clear the line

**Comment:** For the flush volume for epinephrine question, ChatGPT consistently answer correctly, whereas DeepSeek consistently answered incorrectly.

1. **Neonatal resuscitation workshop examinations (Chinese)**
   1. ***Scenario-based question***

**Sample question:**

38周足月新生儿娩出后，已完成初步复苏，你作为主复苏者，观察到患儿无呼吸，助手听诊心率后汇报70次/分，你决定立即给予进行下一步复苏措施，如下描述正确的是？

A .使用自动充气式气囊进行正压通气，氧浓度为21%

B. 你站在患儿右侧，利于操作

C .助手将经皮血氧饱和度探头绑在患儿右脚

D .彻底清理呼吸道，使气道通畅

E .请助手弹患儿足底，刺激呼吸

**Suggested answer:** A

- 1. ***Non-scenario-based question***

**Sample question:**

推荐静脉内1:10000肾上腺素使用后，生理盐水的冲管剂量为：

A. 1ml

B. 0.5ml

C. 3ml

D. 1ml/kg

**Suggested answer:** C

**ChatGPT answer (August 20, September 1):** D. 1ml/kg

**(September 8)**: C. 3ml

**DeepSeek answer (August 20, September 1, September):** C. 3ml

**Comment:** The question regarding flush volume for epinephrine (the same as 2.3 question sample) in Chinese, DeepSeek answered correctly in all three runs, whereas ChatGPT provided incorrect answers in two of the three runs.
