# Supplementary material for: Performance of large language models in neonatal resuscitation assessments versus healthcare providers: an exploratory study
Source: Front Artif Intell. 2026 Jun 25;9:1838877. doi: 10.3389/frai.2026.1838877 (PMC13346239; doi:10.3389/frai.2026.1838877)
Supplement: Supplementary file 2 [file Table_1.docx]

**Supplementary Materials**

**Table 1 Comparison of performance between large language models and healthcare providers (HCPs).**

| **Assessments** | **Overall**  **Accuracy**  **% (n/N)^#^** | ***P* value** | | | |
| --- | --- | --- | --- | --- | --- |
|  |  | **vs. DeepSeek-R1** | **vs. HCPs**  ***Pre-training***  **81.9%**  **(8343/10184)**  **N=268** | | **vs. HCPs**  ***Post-training***  **91.7% (4702/5130)**  **N=135** |
| Neonatal resuscitation workshop examinations (Chinese) | ChatGPT-5  89.5%  (102/114) | 0.492 | | 0.037 | 0.406 |
|  | DeepSeek-R1  92.1%  (105/114) | - | | 0.005 | 0.864 |
| Kahoot quizzes (English) |  | **vs. DeepSeek-R1** | | **vs. HCPs**  **78.3%**  **(4128/5271)**  **N=251** | |
|  | ChatGPT-5  93.7%  (59/63) | 0.011 | | 0.003 | |
|  | DeepSeek-R1  77.8%  (49/63) | - | | 0.918 | |

^#^: n/N represents the percentage of total correct answers over the total number of questions.

**Table 2 Comparison of performance between large language models and healthcare providers by question format, model, and data source.**

| **Model or Data source** | **Overall accuracy, %**  **(n/N)^a^** | **Scenario-based questions, %**  **(n/N)^a^** | **Non- scenario-based**  **Questions, %**  **(n/N)^a^** |
| --- | --- | --- | --- |
| **ChatGPT-5** | | |  |
| Neonatal resuscitation workshop examination | 89.5%  (102/114) | 100%  (42/42) | 83.3%*  (60/72) |
| NRP^®^ 8^th^ Textbook | 96.0%  (216/225) | 96.0%  (60.5/63) | 96.0%  (155.5/162) |
| Kahoot quizzes^a^ | 93.7%  (59/63) | 100%  (3/3) | 93.3%  (56/60) |
| **DeepSeek-R1** | | |  |
| Neonatal resuscitation workshop examination | 92.1%  (105/114) | 95.2%  (40/42) | 87.5%  (63/72) |
| NRP^®^ 8^th^ Textbook | 94.2%  (212/225) | 95.2%  (60/63) | 93.8%  (152/162) |
| Kahoot quizzes^a^ | 77.8%^#^  (49/63) | 100%  (3/3) | 76.7%  (46/60) |
| **Healthcare providers** | | |  |
| Neonatal resuscitation workshop examination  (n=135) | 91.7% (4702/5130) | 92.1%  (1739/1890) | 91.5%  (2963/3240) |
| Kahoot quizzes^b^  (n=251) | 78.3%^#^  (4128/5271) | --b | --b |
|  | | |  |
| **NRP^®^**  **8^th^ Textbook** | **Overall accuracy, %**  **(n/N)^a^** | **Multiple-choice questions, % (n/N)^a^** | **Open-ended questions, %**  **(n/N)^a^** |
| ChatGPT-5 | 96.0%  (216/225) | 97.5%  (193/198) | 85.2%*  (23/27) |
| DeepSeek-R1 | 93.7%  (211/225) | 95.5%  (189/198) | 81.5%*  (22/27) |

^a^:n/N represents the percentage of total correct answers over the total number of questions.

^b^: No comparison was performed because there was only one scenario-based question in the Kahoot quiz.

^#^: P<0.05 for differences between ChatGPT-5 vs. a) DeepSeek-R1, b) healthcare providers using Chi-square or Fisher Exact test (SPSS Statistics v.27).

*: P<0.05 for differences between question formats using Chi-square or Fisher Exact test (SPSS Statistics v.27).
